# A fluorescence assay for detecting amyloid-β using the cytomegalovirus enhancer/promoter

**DOI:** 10.14440/jbm.2017.200

**Published:** 2017-08-07

**Authors:** Zachary M. Carrico, Geneva Le, Roberto Malinow

**Affiliations:** Center for Neural Circuits and Behavior, Departments of Neuroscience and Biology, University of California at San Diego, La Jolla, CA 92093, USA

**Keywords:** amyloid-beta, Alzheimer’s disease, cytomegalovirus, fluorescent reporter, high-throughput

## Abstract

Robust assays for detecting the effects of elevated concentrations of amyloid-β (Aβ) may facilitate Alzheimer’s disease research. An appropriate assay would be high-throughput and enable identification of drugs and genetic mutations that block the effects of Aβ, potentially leading to treatments for Alzheimer’s disease. We discovered that the commonly used cytomegalovirus (CMV) enhancer/promoter is sensitive to the effects of Aβ. By combining the CMV enhancer/promoter with a fluorescent protein, we created a reporter system that produces changes in intracellular fluorescence in response to Aβ. Using hippocampal neurons, we quantified the ability of a CMV-fluorescent protein recombinant reporter to detect both exogenously applied and overexpressed Aβ. This is the first report of a high-throughput enhancer/promoter-based Aβ detection method. The reporter is able to detect the effects of elevated concentrations of Aβ in a high-throughput fashion, providing a new tool for Alzheimer’s disease research and important knowledge about the commonly used CMV enhancer/promoter.

## INTRODUCTION

There is no consensus on the molecular changes that initiate and occur in Alzheimer’s disease [1,2], but the concentration of amyloid-β (Aβ) appears to be important [3-6]. Thus, any technology that can detect the effects of Aβ on neurons may lead to a better understanding of the role of this peptide in Alzheimer’s disease. Many assays have been created to detect Aβ [7-11], but far fewer exist to detect Aβ-induced changes in neurons in a high-throughput system [12]. We discovered that a commonly used fluorescent protein expression system composed of a fluorescent protein under transcriptional control of the cytomegalovirus (CMV) enhancer/promoter is sensitive to the effects of Aβ. In the presence of excess Aβ, there is decreased fluorescence from neurons expressing CMV-fluorescent protein relative to those exposed to an Aβ negative control. This study describes the characterization of this reporter and its use in a 384-well format assay.

## MATERIALS AND METHODS

### General reagents/conditions

Unless otherwise stated, all general chemicals were purchased from Sigma-Aldrich, and all neuroactive reagents from Tocris Bioscience. All procedures involving animals were approved by the Institutional Animal Care and Use Committees of the University of California, San Diego.

### Cultured hippocampal slices

Cultured hippocampal slices were prepared from 6 to 7 day-old Sprague-Dawley rat pups as described previously [13] and used at 6 to 7 days *in vitro* (DIV).

### Single-cell electroporations

Single-cell electroporations were done using 6–7 DIV cultured hippocampal slices according to previously described protocols with minor modifications [14,15]. A Master 8 stimulator (AMPI) generated the pulses to deliver the DNA. 7–10 V square pulses of 1 ms at 200 Hz for 500 ms were applied using ~4 MΩ pipettes filled with 12 ng/µl pCI(GFP) (contains the CMV:GFP reporter) and 350 ng/µl of either pCAG(α-CTF) or pCAG(β-CTF) in Ringer’s solution (5 mM HEPES, 160 mM NaCl, 5.4 mM KCl, 12 mM MgCl_2_, and 2 mM CaCl_2_). Cells were electroporated in a cluster with the pCAG(α-CTF) containing solution, and then ~7–10 cell bodies away, another cluster was electroporated with the pCAG(β-CTF) containing solution.

### Two-photon laser scanning microscopy

A two-photon laser-scanning microscope (Olympus) and mode-locked Ti:sapphire laser (Chameleon, Coherent) tuned to 910 nm were used to image electroporated hippocampal slices. A 40 × 0.8 NA Olympus objective lens was used and the slices were perfused with artificial cerebrospinal fluid (ACSF). Images were acquired every 3 µm. Projections of three stacked sections were analyzed using custom-written software in MatLab (Mathworks).

### Primary neuronal culture preparation and plating

Primary hippocampal neurons were made according to previously described protocols with minor modifications [16,17]. See supplementary information for individual steps. A plate seal (4titude Ltd. part number 4ti-0516/384) was used for 384-well plates to reduce evaporation.

### AAV infection of cultures and reagent addition

Adeno-associated virus (AAV)2(CMV:dsRed) was purchased from the Salk GT3 Core and AAV9(CAG:tdTomato) from the University of North Carolina Gene Therapy and vector core. After 1–3 DIV, half the volume of each 384-plate culture well was removed to leave 45 µl of media. Either 4 µl of 4 × 10^8^ viral genomes/µl AAV2(CMV: dsRed) or AAV9(CAG: tdtomato) was added to the media. At 5–6 DIV, peptides were added to 1 µM final concentration unless otherwise stated. The Aβ1-42 (Aβ42) peptide was purchased from American Peptide Co. (part number 62080) and the scrambled Aβ (same amino acids as Aβ42, but the amino acids are in a random order: Lys-Val-Lys-Gly-Leu-Ile-Asp-Gly-Asp-His-Ile-Gly-Asp-Leu-Val-Tyr-Glu-Phe-Met-Ala-Ser-Asn-Ser-Ala-Ile-Phe-Arg-Glu-Gly-Val-Gly-Ala-Gly-His-Val-His-Val-Ala-Gln-Val-Glu-Phe) was purchased from EMD Millipore (part number AG916). Both were dissolved to 1 mg/ml in 1% NH_4_OH, and diluted in media to neutralize the pH before addition to neurons. Every time the media was replaced, more peptide was added to maintain a constant concentration of 1 µM.

### Aβ42 proteolysis

Proteolyzed Aβ42 was prepared by incubating 1 mg/ml Aβ42 and 250 µg/ml Proteinase K in 1% NH_4_OH for 20 min at 65°C. For the non-proteolyzed Aβ42 control, the Proteinase K was first heated to 99°C for 30 min, and then mixed to a final concentration of 250 µg/ml with 1 mg/ml Aβ42. For proteolyzed Aβ42, the Proteinase K was heat inactivated only after incubation with Aβ42.

### Plate reader fluorescence measurements

A Tecan M1000 Pro plate reader was used to measure the fluorescence of 384-well plates. Multiple-reads per well were averaged. Fluorescence measurements were taken daily starting 2–4 d after AAV reporter infection.

## RESULTS

### Aβ results in decreased fluorescence of the reporter in organotypic hippocampal slices

**Figure 1.**
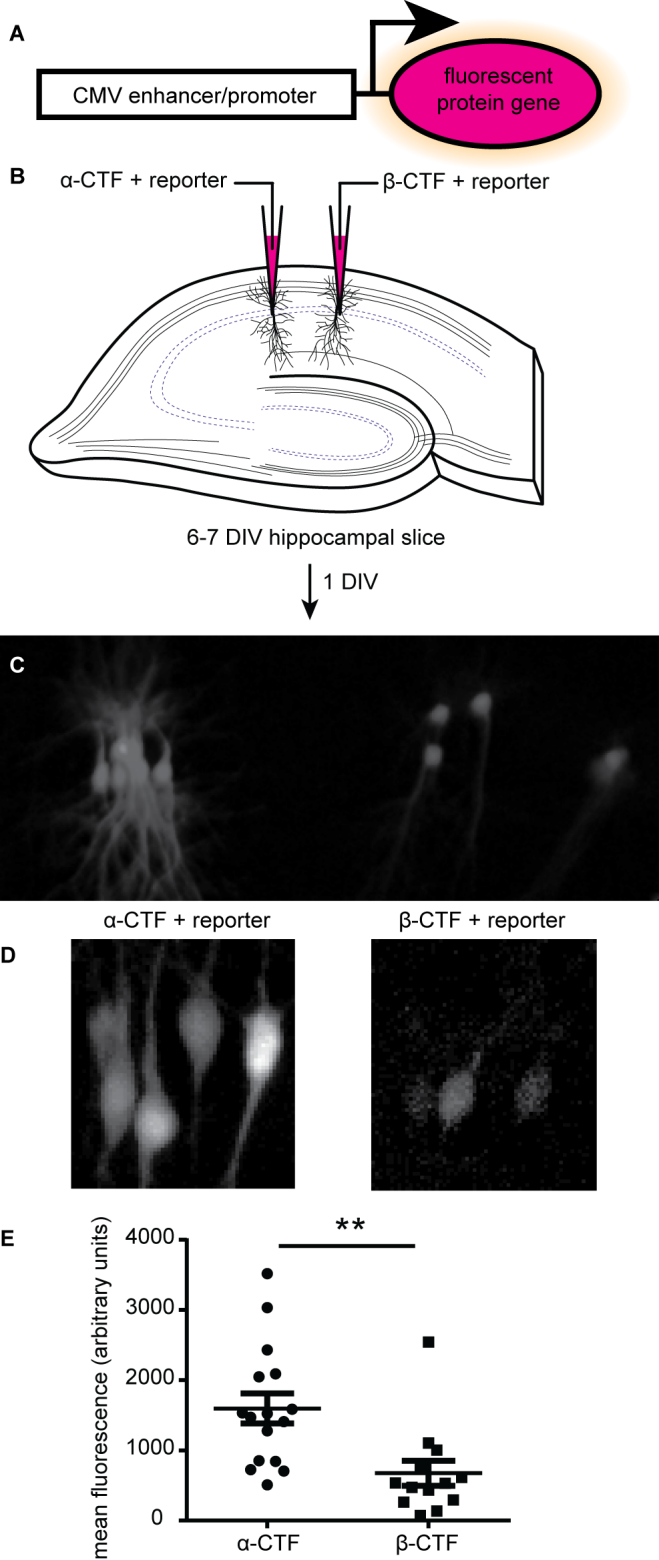
Detecting the effects of amyloid-β on CA1 hippocampal neurons using a CMV:GFP reporter. **A.** Schematic of the reporter. **B.** Depiction of electroporation of hippocampal slices with α-CTF or β-CTF expression plasmids and the reporter plasmid. **C.** Epi-fluorescent microscope image of an electroporated hippocampal slice one day after electroporation. **D.** Two-photon microscope merged z-stack images from the same slice as in (C). **E.** Mean fluorescence from the fluorescent reporter protein (*n* = 16 for α-CTF, or 13 for β-CTF, mean ± SEM, **unpaired *t*-test *P* value < 0.01).

Initial experiments were done using organotypic cultures of rat hippocampi and cultured primary hippocampal neurons were used in all subsequent experiments in a 384-well format. A CMV enhancer/promoter-fluorescent protein reporter (**Fig. 1A**) was used in both hippocampal slice and dissociated neuronal culture. In slice culture, the reporter was co-electroporated with either a plasmid for overexpressing the β- or α-secretase carboxyl terminal products (β-CTF or α-CTF) of amyloid-precursor protein (APP). β-CTF led to production of Aβ, while α-CTF did not and thus served as a negative control. Single-cell electroporation of CA1 neurons [14,15] enables expression of either construct in different neurons of the same hippocampal slice. 16–30 h post-electroporation, two-photon images were acquired (**Fig. 1B**) and the mean fluorescence of the cells was measured (**Fig. 1C**), revealing that the fluorescence level was significantly lower from those neurons overexpressing β-CTF relative to those neurons overexpressing α-CTF.

### Aβ42 peptide results in decreased fluorescence of the reporter in dissociated hippocampal neurons

**Figure 2.**
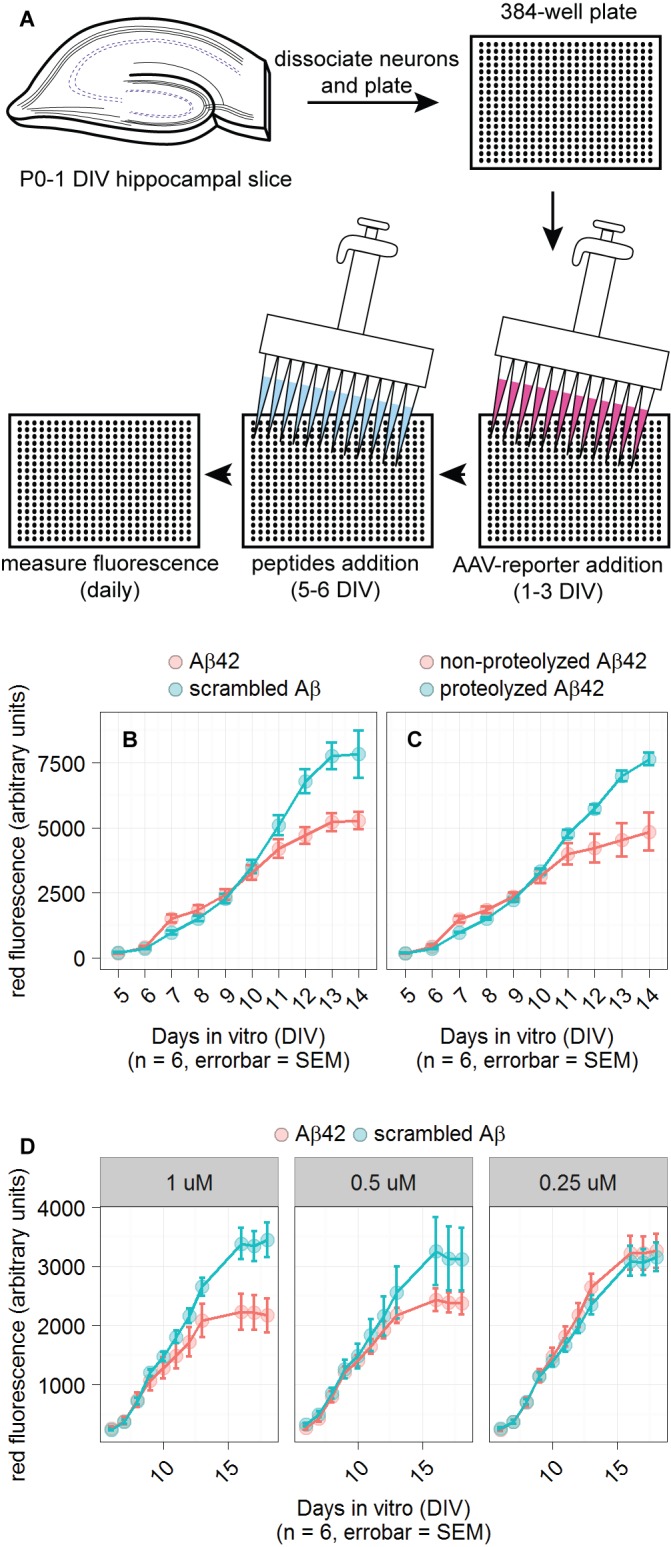
Detection of Aβ42 in primary neuronal cultures. **A.** Schematic of the reporter system in 384-well plates. **B** and **C.** Plots of dsRed reporter fluorescence when cultures were incubated in the designated peptides. Primary neuronal cultures grown in 384-well plates were infected with reporter-containing AAV added at 1 DIV. Indicated peptides were added at 5 DIV, just prior to first plate reader measurement. **D.** Testing three concentrations of peptides: 1, 0.5, and 0.25 µM. NMDA was added to 200 µM at 11 DIV in an unsuccessful attempt to enhance reporter fluorescence. *n* indicates number of wells used for each condition and the error bars represent standard error of the mean (SEM). For all conditions using 1 µM peptide, the difference between reporter fluorescence for Aβ42 and scrambled Aβ conditions had an unpaired *t*-test *P* value < 0.05 on the last day of measurement.

Higher throughput experiments can be achieved with cultures of dissociated hippocampal neurons in 384-well plates. An AAV was used to introduce the CMV-fluorescent protein reporter and synthetic Aβ peptides or scrambled Aβ peptides were added to the media to a final concentration of 1 µM unless stated otherwise. The cultures were infected at 1–3 DIV and fluorescence measurements were taken several days after infection. Peptides were added to the well immediately before the first fluorescence measurement. There was no change in reporter fluorescence after peptide addition within the first day. Fluorescence measurements were taken every 24 h. Two to three days after peptide addition, most Aβ42-incubated cultures had greater fluorescence than scrambled Aβ-incubated slices. This effect was small, but the later decrease in fluorescence in Aβ42-incubated cultures was far larger and consistent. Aβ42 proteolysis with Proteinase K eliminated the effect as shown in **Figure 2C**. We also included non-proteolyzed Aβ42 as a negative control.

The sensitivity of the reporter in terms of Aβ42 titration is shown in **Figure 2D.** The effect of Aβ42 was unambiguous at 1 µM, but was less pronounced at 0.5 µM, and was indistinguishable from scrambled Aβ at 0.25 µM. NMDA (200 µM) was added at 11 DIV to test the hypothesis that it could enhance the reporters’ sensitivity to Aβ, but no enhancement was observed. 1 µM of Aβ42 was used for all the following experiments given its most robust effect.

### Comparing CMV and CAG enhancer/promoters for detecting Aβ42

**Figure 3.**
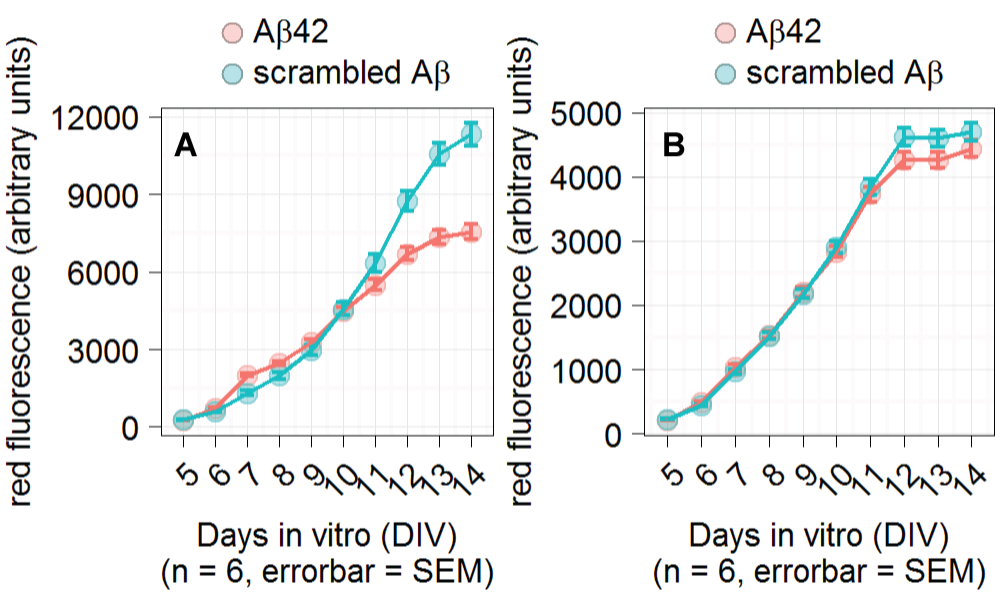
CMV and CAG promoter comparison. Plot of reporter fluorescence. Primary neuronal cultures grown in 384-well plates were infected with reporter-containing AAV virus added at 1 DIV. The indicated peptides were added at 5 DIV and were present throughout the experiment. **A.** The effect of Aβ42 on the CMV promoter expressing the dsRed fluorescent protein. **B.** The effect of Aβ42 on the CAG promoter expressing tdTomato. *n* indicates number of wells used for each condition and the error bars represent standard error of the mean (SEM). Statistical significance for the difference in fluorescence between Aβ42 and scrambled Aβ conditions on the last day of measurement was calculated by an unpaired *t*-test.

The CAG promoter was tested to explore the possibility that other promoters can detect the effects of Aβ42. The CAG promoter contains the CMV enhancer, but uses a chicken-β actin promoter rather than the CMV promoter [18]. **Figure 3** shows a comparison of the two promoters in the presence of Aβ peptides. The effect of Aβ42 was much reduced in neurons expressing the CAG relative to the CMV promoter.

## DISCUSSION

These results show that the commonly used CMV promoter is sensitive to the effects of Aβ42. The effect is robust because it is observed in multiple culture systems, using different transfection methods for overexpressing the reporter, and with different Aβ sources. We initially observed the effects in organotypic hippocampal slices in which CA1 neurons were electroporated to express the reporter with either α- or β-CTF. Cleavage of β-CTF produces Aβ while cleavage of α-CTF produces shorter peptides that are believed to be less toxic than Aβ [11]. As shown in **Figure 1B**, there was far less fluorescence in CA1 neurons overexpressing β-CTF relative to α-CTF, indicating that the reporter was specific to the effects of Aβ relative to a control. To further investigate the reporter and increase experimental throughput, we began using dissociated neuronal cultures plated into 384-well plates. The reporter was introduced into the cells *via* AAV, and synthetic Aβ42 or control peptides were used to investigate Aβ42 sensitivity.

Although the use of 384-well plates allows for more simultaneous replicates than hippocampal slices, the detection of Aβ42 takes longer. In many 384-well cultures, we observed a small difference between Aβ42 and scrambled Aβ two days after peptide addition. This early increase in fluorescence due to Aβ42 was smaller and less consistent than the later decrease in fluorescence, but it has the advantage that it can be observed at an earlier time. Taking advantage of this promoter feature may reduce the assay time by allowing earlier detection of Aβ42. To test that we could eliminate the effect of Aβ42, we proteolyzed Aβ42 with Proteinase K, which resulted in a fluorescence level indistinguishable from scrambled Aβ-incubated cultures (**Fig. 2C**). This was expected because proteolyzed Aβ42 should not initiate the normal Aβ42 signaling pathways.

To understand the mechanism by which Aβ42 reduces reporter fluorescence, we compared the CMV-dependent reporter to a CAG-dependent reporter. CAG uses the same enhancer as CMV, but a different promoter. We found that the CAG reporter system was not significantly affected by Aβ42. This means that either the CMV promoter or the CMV promoter and its enhancer are necessary for detection of Aβ-dependent effects. The CMV promoter is sensitive to silencing *via* methylation, which may be the reason why the CMV-dependent reporter but not CAG-dependent reporter is selectively affected by Aβ42 [19]. In pre-monocytic HL-60 cells, methylation of the CMV promoter completely represses transcription, while CMV enhancer methylation produces minimal effect on transcription [20]. The CAG reporter uses the same enhancer as CMV, but uses a chicken β-actin promoter rather than the CMV promoter. CAG’s β-actin promoter produces more reliable expression across cell lines than the CMV promoter [21,22]. Further experiments will establish the methylation-dependency of these reporters in our culture conditions.

The assay is currently limited to conditions in which Aβ can either be overexpressed or applied exogenously at 1 µM. This concentration is higher than physiological Aβ levels in cerebral-spinal fluid, thus restricting the assay’s application to *in vitro* drug and genetic mutation screens. Potential strategies for expanding the scope of this assay are to replace the fluorescent protein with an enzymatic reporter. For example, luciferase can provide a greater signal-to-noise than fluorescent proteins, potentially enabling more sensitive and faster detection of Aβ.

To summarize, Aβ inhibits florescence signal when the fluorescent protein is under the transcriptional control of the CMV promoter. This finding may be useful in several ways. It can be used in assays to detect Aβ, it provides new avenues for characterization of the signaling pathways initiated by Aβ, and it cautions against using CMV promoter systems for expressing proteins in conditions that might have differing concentrations of Aβ. Future studies will involve exploring the mechanism by which the Aβ sensitivity arises and how this can be used to enhance reporter sensitivity.

## Supplementary Material

Supplementary information **Figure S1.** AAV2(CMV:tdTomato) shows a decrease in fluorescence in response to overexpressed α-CTF relative to β-CTF.
Supplementary information of this article can be found online at
http://www.jbmethods.org/jbm/rt/suppFiles/200.
